# The double-edged sword effect of social comparison orientation on college students’ creativity from the perspective of envy

**DOI:** 10.3389/fpsyg.2026.1816275

**Published:** 2026-06-19

**Authors:** Wenfeng Zheng, Bimei Wang, Lingzhi Cai, Zhao Zhu, Yuyin Jiang

**Affiliations:** 1 Shaanxi Xueqian Normal University, Xi'an, China; 2 Shaanxi Normal University, Xi'an, China

**Keywords:** benign envy, creativity, malicious envy, mastery motivational climate, social comparison orientation

## Abstract

**Introduction:**

While social comparison orientation has been established as an important antecedent to creative performance, existing research mainly focuses on its connection with general creativity (i.e., generating novel and useful ideas that benefit others), largely ignoring its potential link to malevolent creativity (i.e., generating novel ideas intended to harm others). This gap limits a comprehensive understanding of the double-edged motivational consequences of social comparison.

**Methods:**

Drawing on social comparison theory and the dual-pathway model of envy, the current study tests a moderated dual-mediation model in which benign envy and malicious envy mediate the relationship between social comparison orientation and two types of creativity (general vs. malevolent), and a mastery motivational climate moderates these indirect paths.

**Results:**

Three-wave lag survey results show that social comparison orientation positively predicts both benign and malicious envy. Benign envy mediates the relationship between social comparison orientation and general creativity, whereas malicious envy mediates the relationship between social comparison orientation and malevolent creativity. Moderated mediation analyses further indicate that a mastery motivational climate significantly attenuates the indirect effect of social comparison orientation on malevolent creativity via malicious envy, but does not moderate the indirect effect of benign envy on general creativity.

**Discussion:**

These findings highlight the double-edged role of envy in transforming social comparison into distinct creative outcomes, and carry practical implications for educational environments seeking to foster constructive creativity while curbing its harmful forms.

## Introduction

Social comparison is an inherent and ubiquitous feature of human experience, particularly in academic settings where students frequently evaluate their abilities and achievements against those of their peers (Smith et al., 2008). Such comparisons serve as a powerful driving force for motivation and emotional experience (Gold, 1996; McCullough et al., 2004). Although prior research has identified social comparison orientation as a meaningful antecedent of creative performance (e.g., Jiang et al., 2019; Michinov et al., 2015), the literature exhibits a significant imbalance: it has predominantly focused on the link between social comparison and *general creativity*—the generation of novel and useful ideas that benefit oneself or society. This emphasis, however, has largely conceals an equally important parallel pathway—the potential link between social comparison and *malevolent creativity*, defined as the generation of novel ideas intended to intentionally harm others (Cropley et al., 2008; Harris et al., 2013). This oversight limits a comprehensive and dialectical understanding of the motives and consequences of social comparison. It leaves a critical knowledge gap. Specifically, how can the same comparative process drive both socially constructive and socially destructive forms of originality?

The divergence in creative outcomes can be meaningfully illuminated through the lens of envy, a complex social emotion arising from upward social comparisons (Smith and Kim, 2007). Traditionally conceptualized as a unidimensional, hostile construct (i.e., *malicious envy*; Tai et al., 2012), contemporary theory delineates a second, more adaptive pathway: *benign envy* (Lange and Crusius, 2015). Although both forms originate from recognizing another’s superior advantage, benign envy motivates self-improvement and emulation (Crusius and Lange, 2017), whereas malicious envy is characterized by resentment and a desire to diminish the envied other (Krizan and Johar, 2012). This duality suggests that envy may serve as a pivotal psychological mechanism channeling social comparison tendencies into distinct creative paths. Yet critical questions remain unresolved. First, although the situational triggers of envy have been extensively studied, the role of a stable dispositional factor—social comparison orientation—as a precursor to both envy types remains underexplored. Does a high social comparison orientation universally increase susceptibility to envy, or does it selectively predispose individuals toward one emotional outcome? Second, and more centrally, does this envy duality effectively explain the bifurcation of social comparison’s effects on general versus malevolent creativity? Finally, under what conditions are these pathways strengthened or attenuated?

The present study addresses these gaps by proposing and testing a moderated dual-mediation model, grounded in an integration of social comparison theory and the dual-pathway model of envy. We theorize that social comparison orientation serves as a key dispositional antecedent, increasing the likelihood of both benign and malicious envy experiences (Gibbons and Buunk, 1999). These distinct emotional states, in turn, are hypothesized to mediate the relationship between social comparison orientation and divergent creative outcomes: benign envy fostering general creativity through enhanced mastery motivation and cognitive flexibility, while malicious envy fuels malevolent creativity via hostile goal orientation and antagonistic affect. Furthermore, drawing on achievement goal theory, we further identify the perceived mastery motivational climate—an environment emphasizing learning, effort, and personal growth over interpersonal competition (Černe et al., 2014)—as a critical contextual moderator.

We propose that this climate can act as a boundary condition, potentially defusing the destructive pathway from malicious envy to malevolent creativity by rendering harmful actions socially incongruent, while possibly amplifying the constructive pathway from benign envy. Mastery motivational climate directly shapes how individuals appraise upward comparison information. In a high mastery motivational climate, upward comparisons are interpreted as learning opportunities, not as threats to self-worth. This interpretation reduces the hostile activation typical of malicious envy. In contrast, a competitive or performance climate may amplify malicious envy by legitimizing social ranking and zero-sum thinking. Moreover, mastery motivational climate influences the normative appropriateness of envy-driven behaviors. It signals that cooperation, knowledge sharing, and mutual respect are valued. This makes overt hostility socially costly. That is, to simultaneously reinterpret the meaning of social comparison and constrain harmful behavioral expression. Thus, mastery motivational climate is theoretically unique and particularly suitable for examining the boundary conditions of envy to creativity linkages.

**Figure 1:**
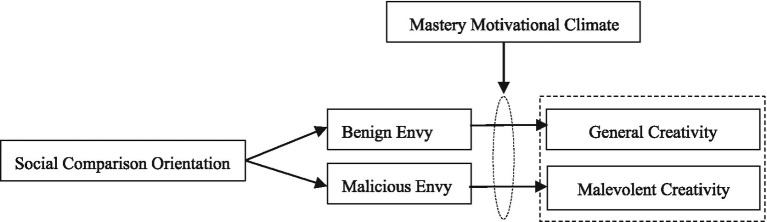
Research model.

By investigating these interconnected relationships, our research aims to make several key contributions. First, it advances the envy literature by clarifying social comparison orientation as a fundamental dispositional driver of its dual forms and by specifying their unique roles as mediating mechanisms. Second, it addresses a significant theoretical gap in creativity research by systematically incorporating its “dark side” into a unified motivational framework alongside general creativity, offering a more complete account of social comparison’s consequences. Third, it enriches our understanding of how achievement contexts shape emotion-behavior linkages by testing the moderating role of the mastery motivational climate. Ultimately, this study seeks to provide a nuanced, integrative framework that explains when and how the ubiquitous process of social comparison translates into creativity that either builds up or tears down, offering valuable theoretical refinement and practical insights for educational environments seeking to harness the positive potential of peer influence while mitigating its risks (Figure 1).

### Review of the literature and formulation of hypothesis

#### Social comparison orientation and envy

Envy is a complex social emotion and a prevalent psychological phenomenon among university student populations. A scientific understanding of its antecedents and consequences constitutes an important line of inquiry within counseling and clinical psychology (Smith et al., 2008). Social psychologists predominantly investigate its influencing factors from a social comparison perspective. Factors such as perceived similarity to the target (Schaubroeck and Lam, 2004), feelings of injustice (Pepper et al., 2015), and attributions of the target’s advantage (e.g., luck vs. effort, Boecker et al., 2022) are established triggers. In contrast, evolutionary psychologists approach envy from the standpoint of resource competition within ancestral environments, positing it as an adaptive psychological response to such competitive scenarios (Hill et al., 2011). Although upward comparisons inherently involve a recognition of inferiority, their emotional sequelae are not uniform. Prior work suggests that such comparisons can trigger either the antagonistic motivation of malicious envy (Reh et al., 2018; Watkins, 2021) or the emulative motivation of benign envy (Liu et al., 2019). Yet, a critical gap remains: few studies have explicitly modeled social comparison orientation as a key dispositional predictor capable of predisposing individuals to experience both forms of envy (Gibbons and Buunk, 1999). Given that a high social comparison orientation individual is more frequently and intensely engaged in comparative self-evaluation, they likely experience a greater volume of envy-evoking stimuli. However, it remains theoretically and empirically unclear whether this heightened reactivity translates equally into both benign and malicious envy pathways, or whether social comparison orientation selectively predisposes individuals toward one emotional outcome.

We argue that social comparison orientation serves as a general amplifier of envy-related experiences. High social comparison orientation individuals more often encounter upward comparisons, which provide two competing interpretations: the gap can be seen as a learning opportunity (leading to benign envy) or as a threat to self-worth (leading to malicious envy). Both interpretations are likely to co-occur, resulting in elevated levels of both envy forms. Therefore, we hypothesize:

*H1*: Social comparison orientation is positively related to benign envy.

*H2*: Social comparison orientation is positively related to malicious envy.

### The mediating role of benign and malicious envy in linking social comparison to creativity

Creativity has long been regarded as a vital driving force for individual growth and organizational development, seemingly carrying an inherent aura of benefiting both the self and society. However, when employed with malicious intent, creativity can also yield negative consequences for individuals and society, revealing its so-called “dark side” (Cropley, 2010). Malevolent creativity epitomizes this dark side, which closely related to general creativity—as both require the generation of novel and useful ideas or solutions—malevolent creativity is distinctly characterized by its underlying motivational goal and the deliberate intent to inflict harm. This critical distinction suggests that the influencing factors for the two forms of creativity likely diverge.

Therefore, grounded in the framework of social comparison theory (Fischer and Manstead, 2008), the current study aims to extend research on the antecedents of creativity by adopting the distinct motivational pathways (a dual-path perspective) of benign envy and malicious envy. It allows for an examination of creativity’s positive aspects (general creativity) while simultaneously exploring its darker manifestations (malevolent creativity). Specifically, benign envy, characterized by a focus on self-improvement and a desire to level up, is posited to fuel general creativity. Empirical evidence supports this proposition, indicating that benign envy strengthens an individual’s mastery-oriented motivation, which in turn boosts divergent thinking and increases perseverance on cognitively demanding tasks, such as those requiring remote associations (Van de Ven et al., 2011). This self-improvement motivation further enhances cognitive flexibility—a key cognitive substrate essential for creative thought. Consequently, through this motivational and cognitive pathway, benign envy serves as a constructive affective driver that facilitates performance in general creativity (Sligte et al., 2011).

Conversely, malicious envy, marked by feelings of resentment and a desire to diminish the envied other’s advantage, is theorized to be a potent catalyst for malevolent creativity. On one hand, the hostile action tendency inherent in malicious envy aiming to “pull the superior other down”, corresponds directly to the harm-inflicting goal intrinsic to malevolent creativity (Tai et al., 2023). Second, malicious envy constitutes a complex hostile affect that includes core components such as anger and hostility (Smith and Kim, 2007). Prior research has independently established that anger, a principal component of this affective complex, can facilitate the generation of malevolent ideas (Cheng et al., 2021). Thus, both the goal-directed orientation and the specific affective composition of malicious envy are posited to enhance an individual’s capacity for malevolent creativity.

Therefore, integrating the above findings with the Hypothesis 1 and 2, benign envy and malicious envy are hypothesized to lead the general comparison tendency produced by a high social comparison orientation into two different creative outcomes. One path leads to constructive, self-centered progress (general creativity), while the other leads to destructive, other-centered harm (malicious creativity). Based on this, we propose the following mediation hypothesis:

*H3*. Social comparison orientation indirectly affects students’ general creativity through benign envy.

*H4*. Social comparison orientation indirectly affects students’ malevolent creativity through malicious envy.

### The moderating role of mastery motivational climate

Beyond dispositional and affective individual differences, prior research has established that motivational climate shapes how individuals interpret and respond to social comparison information. In environments emphasizing learning, effort, and personal growth, people tend to view upward comparisons as opportunities for self-improvement rather than as threats to self-worth (Černe et al., 2014). Such an orientation reduces hostile reactions typically associated with envy. In contrast, performance-oriented or competitive climates amplify social ranking concerns, potentially intensifying malicious envy and its destructive consequences. However, existing studies have not systematically examined whether mastery climate differentially moderates the two envy-to-creativity pathways.

We propose an asymmetric moderation pattern. The rationale stems from the distinct motivational cores of benign and malicious envy. Benign envy is driven by internal self-improvement goals. These intrinsic motives are relatively insensitive to external cues. Even in a low mastery climate, individuals experiencing benign envy still strive to close the gap through effort and learning. Therefore, mastery climate should not significantly alter the link from benign envy to general creativity. In other words, the indirect effect of social comparison orientation on general creativity via benign envy is expected to remain stable across different levels of mastery climate. This leads to an expected null moderation.

In contrast, malicious envy is other-focused. It requires situational permission or justification to translate into harmful action. A high mastery climate raises the social cost of hostility by promoting cooperation, mutual respect, and knowledge sharing (Černe et al., 2014). It constrains the expression of malicious envy into malevolent creativity (Yoshida et al., 2014). A low mastery climate provides fewer normative constraints, allowing malicious envy to more readily manifest as harmful creative behavior. Consequently, the indirect effect of social comparison orientation on malevolent creativity via malicious envy should be weaker when mastery climate is high than when it is low.

Based on the above, we hypothesize:

*H5*: The mastery motivational climate does not moderate the indirect relationship between social comparison orientation and general creativity via benign envy.

*H6*: The mastery motivational climate moderates the indirect relationship between social comparison orientation and malevolent creativity via malicious envy. Specifically, this positive indirect effect will be weaker when the perceived mastery motivational climate is high (vs. low).

## Method

### Participants and procedure

Priori power analysis was conducted by G* power 3.1 (Faul et al., 2009). Assuming the significance level of *α* = 0.05 with 95%(1 − *β*), to detect a medium effect size (*f*^2^ = 0.15), at least 119 participants are required. Considering the possible participants turnover in multi-wave design, we recruited 354 undergraduate students from Chinese universities. Each participant was required to fill out three online questionnaires with a two-week interval in between. We adopted a time-lag design with a 2-week interval between measurement waves to reduce the influence of common method bias (Podsakoff et al., 2003) and provide support for the temporal relationship between variables (Wang et al., 2017). This short interval empirically supports capturing proximal psychological processes while reducing the influence of external confessional events (Dormann and Griffin, 2015).

At Time 1 (*T*1), participants completed questionnaires measuring social comparison orientation and mastery motivational climate, along with demographic information. Time 2 (*T*2; 2 weeks after *T*1), they completed questionnaires measuring benign envy and malicious envy. Time 3 (*T*3; 2 weeks after *T*2), they completed measurements of general creativity and malevolent creativity. To ensure the anonymity of the participants and achieve cross-wave data matching, each participant has created a unique and self-generated identification code. After collecting the data, use these codes to match the responses. After excluding mismatched surveys, the final sample size was 322 (response rate of 90.96%). Among them, 15.80% were freshmen, 37.90% were sophomores, 29.5% were juniors, 12.10% were seniors, 4.70% were graduate students; 57.1% were female, 42.90% were male, and the average age was 20.31 years (SD = 1.82). All participants obtained written informed consent before data collection. The research plan ensures voluntary participation and guarantees the confidentiality of all responses, adhering to established ethical standards and regulations.

### Measurements

The measurement scale was adapted into Chinese according to the reverse translation scheme of Brislin (1980). First, two bilingual doctoral students independently translated the original English items into Chinese. Next, two other bilingual doctoral students, who had not seen the original version, back-translated the Chinese items into English. Any discrepancies between the original and back-translated versions were then reviewed and resolved through discussion among a psychology expert and the four translators. Finally, the Chinese version was piloted with several postgraduate students to confirm the clarity and comprehensibility of all items.

### Social comparison orientation

Social Comparison Orientation was measured at Time 1 using an 11-item scale adapted from Gibbons and Buunk’s (1999) Iowa–Netherlands Comparison Orientation Measure, as revised by Wang et al. (2006). It includes items such as “I always pay a lot of attention to how I do things compared with how others do things”. Responses were recorded on a 5-point Likert scale ranging from 1 (strongly disagree) to 5 (strongly agree), with higher scores indicating a stronger tendency to engage in social comparison. The Cronbach’s *α* was 0.79.

### Benign envy and malicious envy

Benign and Malicious envy were assessed at Time 2 using the 10-item scales developed by Lange and Crusius (2015), including items such as “When I envy others, I focus on how I can become equally successful in the future.” for benign envy, and “If other people have something that I want for myself, I wish to take it away from them.” For malicious envy. Each item was rated on a 7-point Likert scale (1 = strongly disagree, 7 = strongly agree), with higher scores indicating stronger envy of the corresponding type. The Cronbach’s *α* were 0.80 for benign envy and 0.89 for malicious envy.

### Mastery motivational climate

The perceived mastery motivational climate was assessed at Time1 using a 6-item scale adapted from Nerstad et al.’s (2013) measure of motivational climate. A sample item is “In my group/school, one is encouraged to cooperate and exchange thoughts and ideas mutually.” Responses were recorded on a 5-point Likert scale (1 = strongly disagree to 5 = strongly agree). The Cronbach’s *α* was 0.80.

### General creativity

General creativity was assessed using the 13-item scale developed by Zhou and George (2001) at Time 3. A sample item is “I come up with creative solutions to task problems.” Participants rated their agreement with each statement on a 5-point Likert scale ranging from 1 (strongly disagree) to 5 (strongly agree), with higher composite scores indicating a greater level of general creative potential. The Cronbach’s *α* was 0.94.

### Malevolent creativity

We adapted the 13-item malevolent creativity scale by Hao et al. (2016) at Time 3, including hurting people, lying, and playing tricks. A sample item is “How often do you think of ideas on the margins of rules, when conventional ways do not work?” Participants responded on a 5-point Likert scale (1 = never, 5 = usually), with higher scores indicating greater potential for malevolent creative behavior. The Cronbach’s *α* was 0.95.

### Control variables

We controlled the effect of demographic variables (age, gender, grade and major) as suggested in other studies. The results remained consistent regardless of whether these control variables were included, indicating that the hypothesized relationships were not confounded by these factors.

## Results

### Data analysis and common method biases test

To avoid potential common method biases, data were collected anonymously across three time points with items presented in randomized order. Furthermore, Harman’s single-factor test (Podsakoff et al., 2003) with statistical methods to measure the degree of variation in the common method. Our research resulted in nine eigenvalues greater than one for all factors; the variance of the first factor was 18.16% (<40%). Therefore, no serious common method deviations were observed.

### Measurement model

Before testing our hypotheses, we conducted a series of confirmatory factor analyses (CFAs) using Mplus 8.3 to assess the validity of our measurement model. We compared the hypothesized six-factor model (i.e., social comparison orientation, benign envy, malicious envy, mastery motivational climate, general creativity, malevolent creativity) with five alternative models. The results presented in Table 1 show that the baseline model had a good fit for the data (*χ*^2^(1310) = 3,127.264, CFI = 0.911, TLI = 0.901, RMSEA = 0.066, SRMR = 0.058) and fit better (*p* < 0.05) than the other models. Taken together, these tests support the discriminant validity of the constructs.

**Table 1:** Confirmatory factor analyses (*N* = 322).

Model	*χ^2^*	df	RMSEA	SRMR	CFI	TLI
Six-factor model	3,127.264	1,310	0.066	0.058	0.911	0.901
Five-factor model: combine BE and ME	3,585.357	1,315	0.074	0.069	0.873	0.862
Five-factor model: combine GC and MC	6,233.583	1,315	0.108	0.112	0.757	0.734
Three-factor model: combine BE, ME, GC and MC	7,517.236	1,322	0.121	0.135	0.681	0.658
Two-factor model: combine MMC, BE, ME, GC and MC	7,986.575	1,324	0.126	0.142	0.520	0.497

SCO, Social comparison orientation; BE, Benign envy; ME, Malicious envy; MMC, Mastery motivational climate; GC, General creativity; MC, Malevolent creativity; RMSEA, Root mean square error of approximation; SRMR, Standardized root mean square residual; CFI, Comparative fit index; TLI, Tucker–Lewis index.

We also evaluated the discriminant and convergent validity of the measurement model. First, the composite reliability (CR) values ranged from 0.787 to 0.952, meeting Hair et al.’s (2014) criterion of 0.70 or above. Next, the average variance extracted (AVE) values for all variables ranged from 0.53 to 0.69, exceeding the criterion of 0.50 or above (Fornell and Larcker, 1981). Lastly, we assessed discriminant validity using the AVE-SV comparison method (Fornell and Larcker, 1981). As shown in Table 2, the square roots of the AVE values were all higher than the correlations among the constructs, satisfying the discriminant validity criteria (Fornell and Larcker, 1981). Consequently, the results confirmed the measurement model’s discriminant and convergent validity.

**Table 2:** Discriminant validity.

Variables	1	2	3	4	5	6
1 Social comparison orientation	0.524					
2 Mastery motivational climate	0.326	0.643				
3 Benign envy	0.196	0.152	0.673			
4 Malicious envy	0.176	−0.113	0.019	0.784		
5 General creativity	0.231	0.223	0.229	−0.151	0.748	
6 Malevolent creativity	0.059	−0.133	−0.064	0.431	0.072	0.778

The diagonal is AVE values.

### Correlations

The Spearman correlation between descriptive statistics and related variables is demonstrated in Table 3. The results showed that social comparison orientation was related to benign envy (*r* = 0.22, *p* < 0.01) and malicious envy (*r* = 0.23, *p* < 0.01), while benign envy was significantly correlated with general creativity (*r* = 0.23, *p* < 0.01), malicious envy was significantly correlated with malevolent creativity (*r* = 0.43, *p* < 0.01). Thus, these correlations provided initial support for our hypotheses.

**Table 3:** Descriptive statistics and correlations.

Variables	*M*	SD	1	2	3	4	5	6
1. Social comparison orientation (*T*1)	3.16	0.50	(0.79)					
2. Benign envy (*T*2)	4.52	1.00	0.22^**^	(0.80)				
3. Malicious envy (*T*2)	2.92	1.17	0.23^**^	0.02	(0.89)			
4. Mastery motivational climate (*T*1)	3.44	0.60	0.14^*^	0.15^*^	−0.11^*^	(0.80)		
5. General creativity (*T*3)	3.49	0.62	0.12^*^	0.23^**^	−0.15^**^	0.22^**^	(0.94)	
6. Malevolent creativity (*T*3)	2.33	0.87	0.10	−0.06	0.43^**^	−0.13^*^	0.07	(0.95)

*N* = 322, *T*1/2/3 = Time 1/2/3. ^*^p < 0.05. ^**^p < 0.01. ^***^p < 0.001. The diagonal is marked with the Cronbach’s *α*.

### Hypothesis testing

The current study tested our hypotheses with SPSS 26.0 regression analysis. First, we examine the effects of social comparison orientation on both benign envy and malicious envy. As shown in M2 and M4 in Table 4, after controlling for gender, grade, major and age, social comparison orientation demonstrated significant positive effects on both benign envy (*β* = 0.22, *p* < 0.001) and malicious envy (*β* = 0.22, *p* < 0.001). Thus, Hypotheses H1 and H2 were supported.

**Table 4:** Regression results for standardized regression coefficients.

Variables	Benign envy (*T*2)		Malicious envy (*T*2)		General creativity (*T*3)		Malevolent creativity (*T*3)	
	*M*1	*M*2	*M*3	*M*4	*M*5	*M*6	*M*7	*M*8
Gender (*T*1)	0.03	0.04	−0.21^***^	−0.20^***^	0.13^*^	0.10	−0.02^***^	−0.19^***^
Grade (*T*1)	0.01	−0.02	0.04	0.01	0.09	0.13	0.09	0.09
Age (*T*1)	0.03	0.02	0.07	0.05	−0.02	−0.02	−0.02	−0.03
Major (*T*1)	−0.06	−0.01	−0.05	−0.00	−0.09	−0.09	−0.06	−0.07
Social comparison orientation (*T*1)		0.22^***^		0.22^***^	0.07		0.00	
Benign envy (*T*2)					0.21^***^	−0.39		
Malicious envy (*T*2)							0.39^***^	1.08^***^
Mastery motivational climate (*T*1)						−0.28		0.25^*^
Mastery motivational climate × benign envy						0.81^*^		
Mastery motivational climate × malicious envy								−0.74^**^
*F*	0.27	3.46^**^	3.56^**^	6.35^***^	4.46^***^	6.17^***^	15.92^***^	15.32^***^
*R*^2^	0.03	0.05	0.04	0.09	0.08	0.12	0.23	0.26

*N* = 322, ^*^p < 0.05. ^**^p < 0.01. ^***^p < 0.001. All values represent standardized coefficients.

To test the mediation effects (Hypothesis 3 and 4), we employed the SPSS PROCESS macro (Hayes, 2013, Version 3.3) following the analytical procedures outlined by Preacher et al. (2007). Specifically, Model 4 (a simple mediation model; Preacher and Hayes, 2008) was used to estimate the indirect effects of social comparison orientation on general and malevolent creativity via benign and malicious envy. The significance of these indirect effects was determined using bias-corrected bootstrapping with 5,000 resamples to generate 95% confidence intervals. The results (see Table 5) revealed a significant indirect effect of social comparison orientation on general creativity through benign envy (estimate = 0.057, 95% CI [0.013, 0.120]). Similarly, a significant indirect effect was found on malevolent creativity through malicious envy (estimate = 0.041, 95% CI [0.007, 0.080]). Therefore, Hypotheses 3 and 4 were supported.

**Table 5:** Indirect effects.

Model	Effect	SE	Boot LLCI	Boot ULCI
Social comparison orientation → benign envy → general creativity				
Indirect effect	0.057	0.027	0.013	0.120
Moderated mediation effect	0.046	0.028	−0.003	0.106
High SIE (+1SD)	0.078	0.034	0.022	0.150
Low SIE (−1SD)	0.022	0.026	−0.023	0.082
Difference	0.055	0.034	−0.003	0.128
Social comparison orientation → malicious envy → malevolent creativity				
Indirect effect	0.041	0.018	0.007	0.080
Moderated mediation effect	−0.076	0.035	−0.148	−0.011
High SIE (+1SD)	0.105	0.033	0.048	0.176
Low SIE (−1SD)	0.196	0.052	0.099	0.303
Difference	−0.091	0.043	−0.178	−0.014

Building on previous results, we tested the moderated mediation hypotheses by adding an interaction between mastery motivational climate and benign envy (malicious envy) to the model. As presented in Table 5, the indirect effect of social comparison orientation on malevolent creativity via malicious envy varied significantly across different levels of the mastery motivational climate (Δ*B* = −0.091, 95% CI [−0.178, −0.014]). Specifically, this indirect effect was weaker for students who perceived a higher mastery motivational climate. Thus, Hypothesis 6 was supported. In contrast, the indirect effect on general creativity via benign envy did not show significant variation across levels of the mastery motivational climate (Δ*B* = 0.055, 95% CI [−0.003, 0.128]). Therefore, Hypothesis 5 was supported.

**Figure 2:**
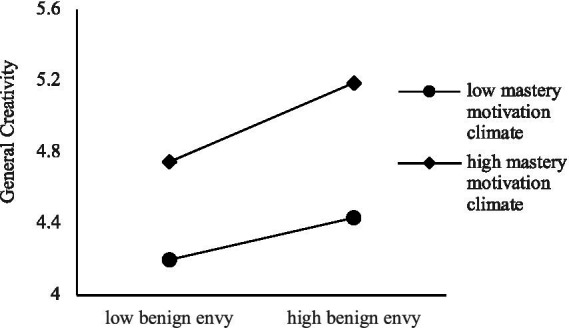
The interactive effect of benign envy and mastery motivational climate on general creativity.

**Figure 3:**
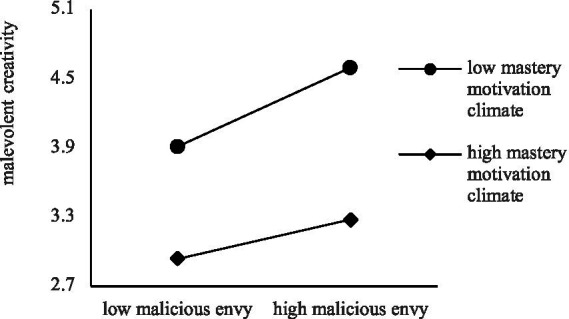
The interactive effect of malicious envy and mastery motivational climate on malevolent creativity.

Finally, to further clarify how the mastery motivational climate moderates the relationship between envy and creativity, the interaction effects were examined. As shown in M6 of Table 4, the interaction term between benign envy and the mastery motivational climate had a significant positive effect on general creativity (*B* = 0.86, *p* < 0.05). Simple slope analysis (see Figure 2) revealed that under a high mastery motivational climate, benign envy had a stronger positive effect on general creativity (effect = 0.181, 95% CI [0.095, 0.267]), whereas under a low mastery motivational climate, this effect was not significant (effect = 0.056, 95% CI [−0.034, 0.145]). Similarly, M8 in Table 4 indicated a significant negative interaction between malicious envy and the mastery motivational climate on malevolent creativity (*B* = −0.74, *p* < 0.01). Following simple slope analysis (see Figure 3), malicious envy showed a stronger positive effect on malevolent creativity under a low mastery motivational climate (*B* = 0.380, 95% CI [0.279, 0.480]). In contrast, under a high mastery motivational climate, the positive effect of malicious envy was attenuated (*B* = 0.204, 95% CI [0.113, 0.295]).

## Discussion

Social comparison orientation positively predicted both benign and malicious envy. Why would the same personality trait give rise to two functionally opposite emotions? According to social comparison theory (Festinger, 1954), upward comparisons provide two competing signals. One signal suggests “I can improve to reach that level”, which triggers benign envy and self-focused striving. The other signal threatens self-worth (“I am inferior”), activating malicious envy and other-focused hostility. Individuals high in SCO encounter these signals more frequently. This finding refines the dual-pathway model of envy (Van de Ven et al., 2009) by identifying a stable dispositional antecedent that simultaneously enables both envy types. It also challenges the traditional view of envy as a unitary hostile construct (Schoeck, 1969).

Benign envy mediated the link from social comparison orientation to general creativity, whereas malicious envy mediated the link to malevolent creativity. This divergence is explained by motivational orientation. Benign envy creates an approach-oriented goal of self-improvement. This state enhances cognitive flexibility and task persistence, which directly support general creativity (Van de Ven et al., 2011; Sligte et al., 2011). Malicious envy, in contrast, generates an avoidance-oriented goal of harming the envied target. It directs cognitive resources toward covert and novel strategies for pulling others down, a process that naturally aligns with malevolent creativity (Cheng et al., 2021). Critically, these two pathways do not cross because their motivational cores are incompatible. By placing general and malevolent creativity as parallel outcomes in one model, our study expands creativity research beyond its traditional focus on positive outcomes (Cropley et al., 2008). It demonstrates that creativity is not inherently virtuous; its expression depends on the emotional and motivational context.

Mastery motivational climate attenuated the indirect effect via malicious envy but did not moderate the benign envy pathway. This asymmetric moderation carries important theoretical implications. Benign envy is driven by internal self-improvement motives, which are relatively insensitive to external climate cues. Even in a low mastery climate, benign envy still promotes general creativity. Malicious envy, however, is other-focused and requires situational permission to manifest as harmful behavior. A high mastery climate signals that cooperation and mutual respect are valued, raising the social cost of hostility (Černe et al., 2014). It thus “defuses” the destructive potential of malicious envy without suppressing the constructive side. This finding integrates achievement goal theory (Ames, 1992) with envy research. It shows that environmental factors do not moderate all emotional pathways equally. The theoretical implication is that boundary conditions should be examined with attention to the motivational core of each emotion.

Taken together, these findings advance the literature in three ways. First, by modeling both envy types simultaneously, we show that social comparison orientation acts as a common antecedent that bifurcates into two distinct emotional-motivational routes. This moves beyond the isolated study of benign or malicious envy. Second, by positioning malevolent creativity alongside general creativity as a co-outcome, we challenge the assumption that creativity is always desirable. Third, by identifying mastery climate as a selective “brake” on the destructive route, we link achievement goal theory to envy and creativity, clarifying how educational environments can foster constructive innovation while mitigating harmful forms.

### Practical implication

The results of this study provide feasible inspirations for educational practice, aiming to leverage the motivational potential of social comparison while reducing its risks. Educators should recognize the dual motivational nature of envy (Van de Ven, 2016) without aiming to suppress it entirely. Rather than attempting to completely eliminate envy, interventions can focus on promoting its functional form. We recommend two low-threshold, curriculum-embedded strategies. First, educators can reframe upward comparisons through simple reflective tasks, such as asking students to identify one specific skill or strategy used by a peer they admire and how they could adapt it (Crusius and Lange, 2017). Second, brief peer-modeling discussions—for example, a five-minute small-group sharing of what they appreciate about a classmate’s work—help normalize benign envy and shift class norms toward emulation rather than resentment. By directing the frustration of envy toward personal mastery goals and self-improvement strategies, these exercises guide students to experience more frequent benign envy, thereby transforming social comparison into a catalyst for general creativity and growth. These strategies require no additional mental health training for educators and fit easily into existing course structures.

Second, mastery motivational climate as a key moderating role provides educators and institutions a powerful and systematic lever. In order to reduce the destructive pathways from social comparison to malicious creativity, it is necessary to consciously cultivate a classroom and institutional atmosphere that does not emphasize normative competition (Kopelman et al., 1990). Teachers can implement teaching strategies that reward effort, progress, cooperation and learning from mistakes. The assessment method that combines personal progress indicators with absolute performance can help students refocus their attention on self-referential growth (Nerstad et al., 2013). By highlighting cooperative norms and psychological safety, such an environment increases the perceived social cost of adversarial behavior, thereby eliminating malicious envy and reducing its transformation into harmful ideas (Chan, 1998). Our research findings particularly indicate that enhancing this climate is especially effective as a protective factor against the development of malicious creative tendencies.

Third, the results emphasize the importance of integrating social and emotional learning with academic guidance to enhance students’ metacognition and self-regulation abilities (Oberle et al., 2016). Projects that enhance emotional granularity—helping students accurately label complex emotions such as envy—and teaching adaptive coping strategies can build resilience against negative impacts related to social comparisons (Barrett et al., 2001; Tugade et al., 2004). In addition, training in moral decision-making and empathy can be combined with creativity training to ensure that students’ rapidly developing creative capabilities are integrated with a strong awareness of the social and ethical impact of their ideas, especially in highly competitive environments. In conclusion, the subtle approach of differentiating types of envy, strategically shaping an atmosphere of achievement, and enhancing students’ personal resources can enable educators to cultivate an ecosystem that promotes productive and general creativity while actively guarding against its potential dark side (malevolent creativity).

### Limitations and future directions

Despite the current study has made contributions in both theory and practice, there are still some research limitations. Future research can further enrich them. First, although we adopted a three-wave, time-lag design to support causal reasoning and mitigate the differences of common methods, the relevance of the study excludes the explicit claim of causality. While the proposed orientation path is based on theory and data support, reciprocity or reverse causality cannot be ruled out (for example, the possibility that creativity influences subsequent envy). Future studies would benefit from experimental manipulations or longitudinal designs with more measurement waves to establish the temporal precedence and dynamic interplay among the core constructs. Second, the reliance on self-reported measures, despite their established validity, introduces the potential for common method bias (Podsakoff et al., 2003) and shared variance, particularly for socially sensitive constructs like malicious envy and malevolent creativity. Although procedural remedies (e.g., temporal separation, anonymity) and statistical checks were applied, future research could incorporate multi-source data, such as peer or teacher ratings of student creativity, or employ objective behavioral tasks to assess creative performance, thereby strengthening the validity of the findings. Third, the generalizability of our findings may be constrained by the cultural and demographic characteristics of the sample, which consisted exclusively of Chinese university students. Cultural norms significantly shape the experience and expression of envy (e.g., De Zoysa et al., 2021; Lange and Crusius, 2015), as well as the prevailing classroom climate. Replicating this model in more diverse cultural contexts and across different educational levels (e.g., high school, workplace settings) would test the boundary conditions of the proposed relationships and enhance their external validity. Finally, our moderated mediation model, while informative, captures only a portion of the complex nomological network surrounding envy and creativity. The study focused on a mastery motivational climate as a contextual moderator; however, other interpersonal interaction factors, such as conflict (Hülsheger et al., 2009) or passion (Chen et al., 2015), may also critically shape the envy-to-creativity pathways. In addition, individual differences like core self-evaluation (Su et al., 2024) or moral identity (Roberts, 2003) could serve as important personal boundary conditions, determining whether upward comparisons trigger benign or malicious envy. Future study could investigate these moderators in many aspects would provide a more detailed understanding.

## Conclusion

By integrating social comparison theory with the dual-pathway model of envy, the current study showed that social comparison orientation has a positive relationship with both benign envy and malicious envy, which in turn mediate the effects on general creativity and malevolent creativity, respectively. Furthermore, the mastery motivational climate attenuates the pathway from malicious envy to malevolent creativity. These findings provide novel and meaningful insights for fostering adaptive creativity while mitigating its harmful counterparts in educational environment.

## Data Availability

The original contributions presented in the study are included in the article/supplementary material, further inquiries can be directed to the corresponding authors.
